# Accuracy of sentinel lymph node mapping in detecting occult neck metastasis in papillary thyroid carcinoma

**DOI:** 10.20945/2359-3997000000038

**Published:** 2018-05-07

**Authors:** Jose Higino Steck, Elaine Stabenow, Gustavo Baldove Bettoni, Samuel Steck, Claudio Roberto Cernea

**Affiliations:** 1 Clínica Onccape Serviço de Cirurgia de Cabeça e Pescoço Campinas SP Brasil Clínica Onccape, Serviço de Cirurgia de Cabeça e Pescoço, Campinas, SP, Brasil; 2 Universidade de São Paulo Universidade de São Paulo Faculdade de Medicina Hospital das Clínicas São Paulo SP Brasil Hospital das Clínicas, Faculdade de Medicina da Universidade de São Paulo (HCFMUSP), Serviço de Cirurgia de Cabeça e Pescoço, São Paulo, SP, Brasil

**Keywords:** Thyroid gland, papillary cancer, sentinel lymph node biopsy, lymphatic metastases, neck dissection

## Abstract

**Objectives:**

The objectives of this study were to evaluate the following: 1) the accuracy of sentinel lymph node mapping (SLNM) in detecting metastasis in papillary thyroid carcinoma (PTC), and 2) if SLNM could modify the American Joint Committee on Cancer (AJCC) staging of previous cN0 PTC patients.

**Subjects and methods:**

Forty SLNM were performed prospectively in 38 consecutive cN0 PTC patients, with total thyroidectomy and elective compartment neck dissection (CND). The results of SLNM were compared with CND pathological findings to verify the accuracy of sentinel SLNM.

**Results:**

The mean patients’ follow-up was 36 months. A total of 133 SLN were found at levels VI, II, III and IV. The SLN was identified in 95% of the patients with one false negative, 95% sensitivity, a 94% negative predictive value and 97% accuracy. The SLNM upstaging from cN0 to pN+ was 49%, and to stages III and IVa, it was 21%.

**Conclusions:**

For this series of cN0 PTC patients: 1) SLNM accuracy was 97%, and 2) SLNM upstaging from cN0 to pN+ was 49%, whereas to stages III and IVa, it was 21%.

## INTRODUCTION

Papillary thyroid carcinoma (PTC) is the most common endocrine neoplasm and has shown a global increase in its prevalence in the past three decades (1). The incidence of lymphatic metastasis in PTC at initial diagnosis varies from 30% to 90% in different reports (2,3), and they are more commonly found in the central compartment. However, most lymph nodes harbor micrometastasis, which is not detectable clinically or radiologically. A controversy exists in the literature regarding the necessity of elective or prophylactic central compartment neck dissection (CND) in patients classified as cN0 (neck clinically without metastasis) PTC, according to the American Joint Committee on Cancer (AJCC) (4,5). Uncertainty about the real impact of this metastasis in the prognosis is one of the reasons for this controversy. In addition, an increased risk of postoperative complications is related to elective CND, such as hypoparathyroidism (3). Therefore, a less invasive method, such as sentinel lymph node mapping (SLNM), may be used to evaluate the true pathologic status of clinically normal lymph nodes in these patients.

SLNM has been widely used in patients with cutaneous melanoma, breast cancer and other malignant solid neoplasms, to stage them and to indicate lymph node dissection only in cases with histologically proven metastasis. The SLNM has the potential of avoiding CND and its risks to the neck with no metastasis (pN0). Even with no previous indication of elective CND, SLNM can be used to stage the neck and to suggest further treatment with radioiodine therapy if the clinical team considers this to be necessary. SLNM has potential advantages when compared with elective CND: It helps one to avoid the morbidity of CND in negative necks and to better stage the lateral compartment when lateral drainage is present.

The objectives of this study were to verify the accuracy of SLNM in detecting occult neck lymph node metastasis in PTC, and to evaluate if the SLNM could modify the stage grouping of patients with PTC cN0.

## SUBJECTS AND METHODS

After institutional review board approval and written informed consent were obtained, 38 consecutive patients with thyroid nodes were evaluated prospectively. Two of them had bilateral thyroid nodes, so were performed a total of 40 SLNM. The inclusion criteria were as follows: fine needle aspiration (FNA) cytology classified as Bethesda V or VI, clinically N0, evaluated with ultrasonography, at Mario Gatti Hospital in Campinas, Brazil, between 2010 and 2015. The exclusion criteria were as follows: 1) previous neck surgery or radiotherapy, 2) final histopathological exams without thyroid neoplasm confirmation and 3) patients without radioisotope lymphatic drainage.

All 38 patients underwent the preoperative ultrasound-guided intratumoral injection of a radioisotope (99mTc-phytate). In 23% of procedures (9/40 procedures), the injection was given the evening before surgery, and the other 77% of procedures were injected in the operating room. The volume of the isotope injected ranged from 0.02 to 0.10 ml, with higher activity for those injected the evening before surgery (400 to 1200 microCi).

Total thyroidectomy was performed before the SLNM in all cases to eliminate shine through effect and to facilitate SLN identification. Following total thyroidectomy, the radio guided SLNM was performed following the standard technique adopted for melanoma. The central and lateral compartments were scanned bilaterally with gamma probe Neoprobe 2000^®^ (Neoprobe Corporation, Cincinnati, OH, USA); a collimator was used to obtain a little thickness (7.5 mm) of the detection center. The contralateral side was used to quantify the background (BG) radioactivity. Next, nodes with three times or more radioactivity than the BG were located and removed. Other nodes were removed if they reached 10% of the hottest SLN count (10% rule) (6). The average time course of mapping was 24.5 minutes, with the time range stretching from six to 50 minutes.

After the completion of SLNM, elective conventional CND was performed.

The final histopathological report of the SLN was compared with the final pathology report of the specimen of the CND, to verify if SLNM was able to detect occult neck lymph node metastasis in cases with PTC.

The SLN analysis was conducted through serial sections in paraffin blocks and hematoxylin and eosin staining. After this analysis, patients were classified as the TNM system from the AJCC (5).

To verify the effectiveness of SLNM in detecting lymphatic metastasis in PTC, the indexes used to evaluate the diagnostic tests were calculated: quantity of false negatives and positives, sensitivity, specificity, accuracy, and positive and negative predictive values. The statistical power was calculated using program G*Power 3.0.10, 2008, Universitat Kiel, Germany.

For a comparison between the averages of two parametric sample populations, Student's t-test was used (7,8), and for a comparison of the frequency, the Fisher's exact test (9,10) with IBM SPSS^®^ Statistics 19.0.0, 2010; Illinois, USA, was employed. Statistical significance was defined as p ≤ 0.05.

## RESULTS

**Figure 1:**
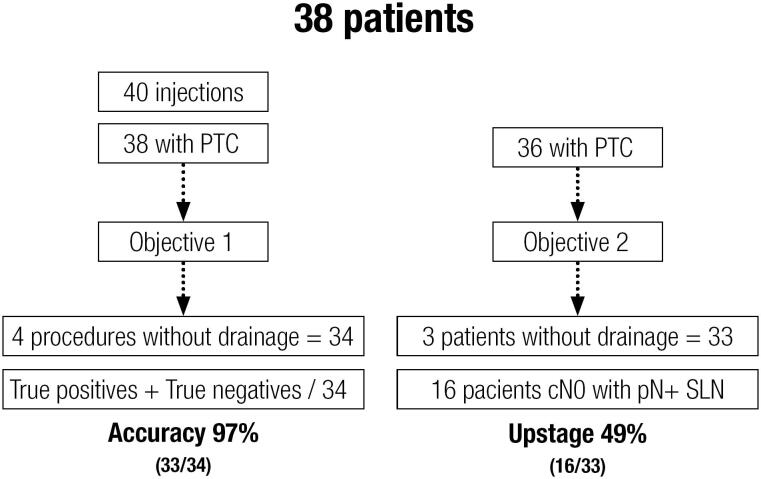
Flowchart summarizing the casuistic, objectives and results. PTC: papillary thyroid carcinoma.

A flowchart revealing the objectives and results is displayed in Figure 1.

Tc99 injection was performed in 38 patients; females were 90% (34/38) of them in a proportion of 9:1. The mean age of the casuistic was 44 ± 15 years (range: 20 – 76 years).

The FNA biopsy of 40 thyroid nodes revealed that 72% (29/40) had malignant findings (Bethesda VI), and 28% (11/40) were suspicious for malignancy (Bethesda V). The histopathological final diagnosis confirmed papillary thyroid carcinoma in 95% (38/40) of biopsied thyroid nodes. The two cases without thyroid neoplasm were excluded for the metastasis analyses.

The primary tumor mean diameter was 14 ± 9 mm (range: 3 – 43 mm). The 38 primary tumors were classified as pT1a: 42% (16/38), pT1b: 24% (9/38), pT2: 8% (3/38), pT3: 24% (9/38) and pT4a: 2% (1/38).

In 4/40 (10%) of the SLN scintigraphy procedures, no Tc99 lymphatic drainage occurred, so it was not possible to localize the SLN. These four procedures were excluded. In addition, one patient presented no drainage at the left-side node but exhibited drainage from the right-side injection. Thus, we had a total of 36 feasible SLNM in 35 patients. The mean size of these four tumors was 33 ± 9 mm compared with 11 ± 6 mm of the tumors with Tc99 drainage (Student's t-test, p = 0.0001). The three largest tumors of the sample did not have any Tc99 drainage. Two of these four tumors without drainage presented positive CND and two negative without recurrence with 39 and 43 months of follow-up.

Considering the 36 procedures with radioisotope drainage, two SLNM did not present any lymphoid tissue at the histologic evaluation. Therefore, the SLN was properly identified in 34 of 36 (95%) procedures in which SLNM was feasible. Thus, 95% of SLNM were effective.

In 35/36 (97%) of the feasible SLNM, SLN was found in the central neck, and in 12/36 (33%), it was found in the lateral neck. One case revealed only a lateral SLN. In 4/36 (11%) of feasible mappings, SLN was at level II, 9/36 (25%) at level III and 2/36 (6%) at level IV.

The mean number of SLN identified in each procedure was 4 ± 2 nodes and at the central neck the mean number was 3 ± 2 lymph nodes (range: 1 to 8 nodes).

Considering cases with feasible SLNM and with confirmed PTC (34 procedures), 16/34 (47%) had a metastatic SLN in the central neck, and 4/12 (33%) of the SLNMs had drainage to the lateral neck. Considering procedures with positive SLN in the central neck, only 9/16 (56%) presented other positive nodes in the CND specimen, and 3/16 (19%) presented node metastases detected in the lateral compartment as well. One patient presented skip metastasis (positive lateral neck lymph node with no positive central compartment lymph node).

Surgical complications were as follows: vocal fold paralysis in 4/76 (5%) laryngeal nerves at risk, 3/76 (4%) with temporary paralysis and 1/76 (1%) with definitive paralysis. All paralyses were unilateral. Transient hypoparathyroidism occurred in 3/38 (8%) patients, and definitive hypoparathyroidism was observed in 1/38 patients (3%).

Resected parathyroid glands were detected at the final pathology report: only 9% (3/32) were found on SLNM specimens, whereas the other 91% were found on CND specimens. Thus, the risk of inadvertently resected parathyroid glands was 10 times higher in CND specimens compared with SLNM specimens.

The mean follow-up period was 36 ± 13 months (range: 3 – 56 months). A minimum follow-up of 24 months was achieved in 83% (30/36) of the cases with PTC.

The specific disease survival was 100%, and the disease-free survival was 89 ± 11%. Only one patient developed recurrence in the contralateral neck after 46 months.

### SLNM accuracy

The comparison between the SLNM and the final pathologic classification of cervical lymph nodes is shown in Table 1. For these analyses, were excluded the two procedures without confirmed papillary carcinoma and the four SLNM without radioisotope lymphatic drainage.

**Table 1:** Comparison between sentinel lymph node mapping results with the final pathologic classification of cervical lymph nodes in patients treated for papillary thyroid carcinoma

pN status	SLN mapping pathologic status		Total
	Positive**	Negative**	
pN positive	17 (94%)	1 (6%)	18 (100%)
pN negative	0 (0%)	16 (100%)	16 (100%)
Total	17 (50%)	17 (50%)	34 (100%)

SLN: sentinel lymph node; pN: pathological status of the cervical lymph nodes.

** Fisher's exact test: p = 0.0001.

The values used to evaluate the diagnostic tests were calculated to determine the diagnostic ability of the SNLM to detect neck node metastases in PTC (Table 2). The statistical power was 95%.

**Table 2:** Diagnostic test evaluation values of the sentinel lymph node mapping to detect lymph node metastasis of papillary thyroid carcinoma

	Values
False positive	0 (0%)
False negative	1 (3%)
Sensitivity	94%
Specificity	100%
Predictive positive value	100%
Predictive negative value	94%
Accuracy	97%

The false-negative case revealed no lymphoid tissue in the histologic SLN specimen. However, microscopically positive metastatic nodes were detected in the CND pathology report (1/5). This case involved a 42-year-old man, with a 10mm PTC macroscopically extended to the trachea, classified as T3N1aM0. He underwent radioiodine therapy and was followed for 47 months. He presented recurrence at the lateral neck nodes 46 months after surgery and was treated via neck dissection.

**Figure 2:**
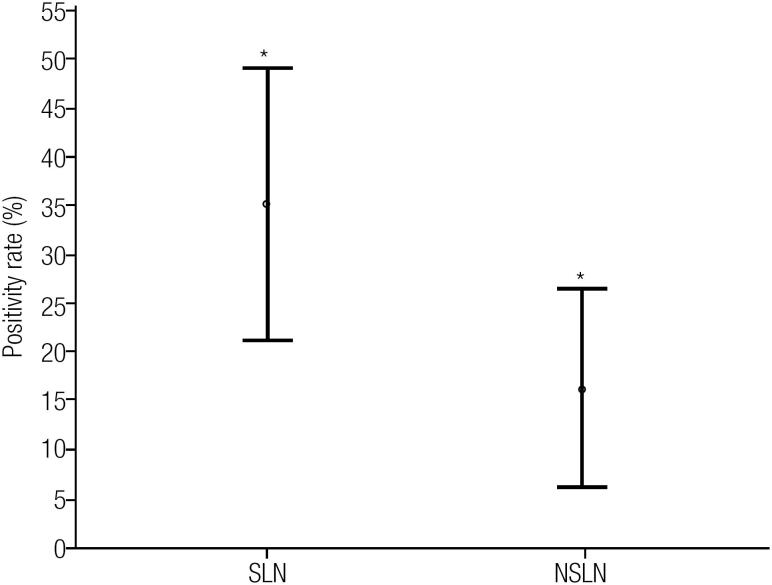
Comparison between the mean of sentinel lymph node positivity rate of papillary thyroid carcinoma metastasis (and 95% confidence interval) and the non-sentinel lymph node positivity rate (Student's *t*-test, p = 0.031).

To determine the density of the SLN positivity, the SLN positivity ratio (PR) was idealized and calculated by dividing the number of positive SLN by the total number of SLN. The PR was obtained for non-SLN too. The mean SLN PR (35 ± 7%) was higher compared with the non-SLN PR (16 ± 5%) (p = 0.031) (Figure 2).

### Upstaging after SLNM

For this analysis, we considered the number of patients instead of procedures. Of the 38 initial patients, the following were excluded: two without confirmed carcinoma and three without any radioisotope lymphatic drainage.

As all patients included in this study were classified as clinically N0, SLNM upstaging was 49% (16/33). Final pN classification, based only upon the SLNM, was as follows: 17/33 (52%) pN0; 12/33 (36%) pN1a and 4/33 (12%) pN1b. For CND specimens, the final N classifications were 50% (18/36) pN0 and 50% (14/36) pN1.

The mean age of the patients with a positive SLN was 38 ± 14 years compared with 49 ± 13 years for those with negative SLN (Student's t-test, p = 0.015). A negative correlation was found between age and positive SLN number (Pearson's correlation coefficient = −0.34, p = 0.039).

The postoperative TNM staging of the 36 patients with PTC was: stage I, 72% (26/36); stage II, 3% (1/36); stage III, 8% (3/36) and stage IVA, 17% (6/36).

## DISCUSSION

PTC has a high frequency of occult lymph node metastases in various series (2), confirmed in the present series as 50%. Although still a subject of controversy, the prognostic value of lymph node metastases was revealed in some recent studies, and it has been taken into consideration for determining the AJCC's TNM staging system, which designates the mortality risk, and the American Thyroid Association risk stratification, which states the recurrence risk (5,11,12). Therefore, there has been an increasing interest in determining the lymph node involvement in PTC to appropriately stage the patient, with the goal being to decrease the locoregional recurrence rate (12). Ultrasonography of the neck is considered the best method for staging the neck before surgery, but the sensitivity for detecting metastasis in level VI when the thyroid gland is still present is low, 35 to 50% (13,14). The most sensitive method for determining the presence of positive nodes in the central compartment in PTC cN0 is CND, which can be performed electively at the same time as thyroidectomy (15,16). Some meta-analysis indicated a higher recurrence-free survival rate in patients undergoing CND (17). However, a controversy exists in the literature regarding whether the elective CND must be indicated in all patients with PTC, due to increased complications, especially transient hypoparathyroidism. Moreover, not all PTC patients receive treatment from experienced surgeons, which is essential for reducing the complication rate. In fact, significant discrepancies can be found among the various evidence-based guidelines of the important societies of experts involved in the treatment of PTC. Some suggest elective CND only for advanced cases of PTC (T3 and T4), whereas others recommend it for all cases (11,18,19). In the present study, CND was performed in all patients after SLNM, to compare the SLN status with the remaining CND and to meticulously evaluate its accuracy.

In the present study, SLNM provided the same information for lymph node staging as CND did. Thus SLNM could adequately select patients who would benefit from CND. If the SLN was histologically negative, CND did not need to be performed. On the other hand, if the SLN was positive, the indication for the CND was no longer elective (or prophylactic) but rather therapeutic. This was an initial study whose aim was to compare the sentinel lymph node content with the histological examination of the neck dissection, the gold standard method for detecting the presence of neck metastasis, to establish SLNM feasibility in the neck. We will continue the study with an intraoperative frozen section investigation of the SLN. Thus, in the same operative procedure, we will be able to perform neck dissection only for patients with positive sentinel lymph node.

Immunohistochemistry could be used for a post-operative micrometastasis search, although the impact of these micrometastases on the prognosis is not well established.

Some features of the central compartment of the neck can lead to greater technical difficulty for SLNM, such as the small sizes of the lymph nodes in the region (20), and the proximity of the site of radioisotope injection in the primary tumor. Therefore, the first procedure must be thyroidectomy, to reduce background radioactivity, thus preventing the so-called shine-through effect of the injection site, which could hide nearby lymph nodes.

Three meta-analyses (21–23) evaluated the SLNM technique for PTC. The vast majority of studies employed only blue dye to map the SLN. In the two largest and most recent meta-analyses, only six studies using the radioisotope were included (22,23). The injection site of the radioisotope, intra or peritumoral, did not have any influence on the SLN detection rate (23). All meta-analyses concluded that using only the blue dye would be less effective for detecting the SLN than the radioisotope, alone or in combination with the blue dye. The techniques using the radioisotope had higher detection rates (83% with the blue dye compared with 96 to 98% with the radioisotope) (21). In the present study with the use of the radioisotope, the SLN was detected in 94.5% of SLNM.

After radioisotope injection, 4/40 SLNM did not demonstrate any lymphatic drainage. These four patients had an average diameter of the primary tumor that was three times larger than that of those with lymphatic drainage. Therefore, although larger primary tumors usually have a higher risk of metastasis, in this series, they had a greater chance of drainage failure. It is possible that in some of these tumors, the lymphatic vessels were already blocked via neoplastic cell invasion, impairing the migration of the radioisotope. SLN drainage difficulty and the increased risk of false negatives have been reported when the SLNM is used for locally advanced tumors from other locations, such as oral cancer (24).

### SLN accuracy

In 97% of the SLNM, the SLN was located in the central compartment and in lateral compartment in 33%. In one case, drainage to the lateral compartment was found without concomitant drainage to the central compartment. Another patient had central and lateral drainage, but metastasis was confirmed only in the lateral SLN. Thus, another advantage of SLNM is to detect these unusual drainage patterns to the lateral compartment at the time of the initial treatment (skip metastases) (25).

In half of the SLNM, proven SLN metastasis was discovered. Additional metastases to non-sentinel lymph nodes were found in 56% of CND specimens.

One false negative SLN (negative SLN with other positive lymph nodes in the central compartment) existed. In this patient, the hot SLN contained, in fact, only thymus fragments in the final pathology report. This was the only patient of the series who had lymph node recurrence on the contralateral lateral neck.

Sensitivity was 94.4% with a specificity of 100%, the positive predictive value was 100% and the negative predictive value of 94.1%. The accuracy of SLNM in detecting lymph node metastases in PTC was 97.1%. These results are comparable to the best data published in the literature, although few authors who reported the use of the radioisotope have been able to calculate the accuracy of the method, as the gold standard of pN status of the whole CND specimen was not available in all cases. In the meta-analysis of Balasubramanian and Harrison (22), which analyzed 24 studies, the SLNM accuracy was evaluated only in 15. Of these, however, 13 used only the blue dye, and the SLNM sensitivity of this group was 91.6%, with 100% specificity, 95.8% accuracy and a false negative rate of 7.7%. Two meta-analyses evaluated the use of the radioisotope, with or without blue dye, with a sensitivity of 67%, specificity of 100%, accuracy of 88% and false negative of 16%. Another study of 25 patients featured a sensitivity of 100%, specificity of 100%, accuracy of 100% and false negative of 0%. This variability of the accuracy can stem from the use of various techniques and the SLNM learning curve in thyroid surgery. In the present study, the team's experience with the use of SLNM in other cancers, combined with experience in thyroid surgery, may have led to the good accuracy level.

In this series, frozen sections were not performed because, by protocol, all patients would be subjected to CND anyway. The authors plan to evaluate the efficacy of SLNM with frozen sections to determine the need for CND in the same surgery. In the literature, frozen sections present a false negative rate of 5-12% in detecting lymph node metastases in PTC (22).

SLN's positive ratio (PR) was higher (35%) than the non-SLN PR (16%) was, suggesting that the concept of SLN would apply to the PTC similarly as in other solid tumors. When metastasis is present, they would be more likely to be found in the SLN than in any other neck node.

### Upstaging

The SLNM could improve thyroid cancer staging, as it had better sensitivity and accuracy compared with ultrasound. During the surgery, the accuracy of SLNM in detecting lymph node metastasis was the same as that of the CND, and it was better than surgeon-performed intraoperative palpation (22,26). The presence of positive SLN metastasis in the central compartment increased the cN0 patient's staging to pN1a (39%). The SLNM was able to make the diagnosis of occult metastases in the lateral compartment, which is an advantage compared with elective CND, which evaluates only the central compartment, with the potential to increase the staging from cN0 to pN1b when the lateral SLN is positive (11%).

In the present study, if only CND had been analyzed, the lymph node upstaging would be 50%, similar to SLNM upstaging (49%). The CND staging, however, would be in the central compartment only (pN1a). Without SLNM, information on the lateral compartment would have been lost in this series in 11% of the procedures. Thus, we considered that the SLNM was at least as effective as the CND was for properly staging the central neck, with the advantage of providing information about the lateral compartment.

In this series, it was not possible to assess the complications of the SLNM procedure because CND was performed in all patients. However, at least in theory, the main complication of CND, which is hypoparathyroidism, would be reduced in SLNM. In fact, only three out of a total of 32 (9.4%) parathyroid glands were removed accidentally via SLNM, whereas the other (90.6%) were removed in the CND specimen, a 9.6 times greater risk of resection in the CND versus SLNM.

This upstaging in the lymph node status could be particularly important in patients older than 45 years old. In this age group, the AJCC staging would be III or IV in the presence of metastases, maybe with a negative prognostic impact (27). In the present series, the upstaging to stage III or IV in this age group was observed in 21% of the cases. Interestingly, occult metastases to SLN were found more frequently among young patients; yet, in this age group, they do not affect the AJCC staging.

In conclusion, this study revealed that the SLN concept applies to PTC just as in other solid tumors. Larger tumors were less likely to have lymphatic drainage. The accuracy of SLNM in detecting cervical lymph node metastases in PTC was 97%. The sensitivity was 94.4%, with a specificity of 100%, a positive predictive value of 100% and a negative predictive value of 94.1%. SLNM upstaging from cN0 to pN+ PTC patients was 49%, and to AJCC stages III or IVa, upstaging made up 21% of the cases. The accuracy was comparable to CND but with the advantage of also staging the lateral neck compartment and theoretically with less risk of hypoparathyroidism than the CND.
